# Exosomal circRNA BTG2 derived from RBP-J overexpressed-macrophages inhibits glioma progression via miR-25-3p/PTEN

**DOI:** 10.1038/s41419-022-04908-4

**Published:** 2022-05-28

**Authors:** Lei Shi, Ying Cao, Wei Yuan, Jun Guo, Guan Sun

**Affiliations:** 1 Department of Neurosurgery, The First People’s Hospital of Kunshan Affiliated with Jiangsu University, Suzhou, Jiangsu P. R. China; 2 Department of Ear-Nose-Throat, The Second People’s Hospital of Huai’an, Huai’an Affiliated Hospital of Xuzhou Medical University, Huai’an, P. R. China; 3 Department of Neurosurgery, The Yancheng Clinical College of Xuzhou Medical University, The First people’s Hospital of Yancheng, Yancheng, P. R. China; 4 Department of Central Laboratory, Yancheng Medical Research Center of Nanjing University Medical School, Yancheng, P. R. China

**Keywords:** CNS cancer, CNS cancer

## Abstract

Macrophage-derived exosomes (Mφ-Exos) are involved in tumor progression, but its role in glioma is not fully understood. *RBP-J* is related to macrophage activation. In this study, we assess the role of exosomes derived from *RBP-J*-overexpressed macrophages (*RBP-J* OE Mφ-Exos) in glioma. The circular RNA (circRNA) profiles in *RBP-J* OE Mφ-Exos and THP-1-like macrophages (WT Mφ)-Exos were evaluated using circRNA microarray. Then the functions of Mφ-Exo-circRNA in glioma cells were assessed *via* CCK-8, EdU, Transwell invasion, and nude mouse assays. Besides, luciferase reporter assay, RNA immunoprecipitation, and Pearson’s correlation analysis were adopted to confirm interactions. We found that circRNA BTG (*circBTG2*) is upregulated in *RBP-J* OE Mφ-Exos compared to WT Mφ-Exos. *RBP-J* OE Mφ-Exos co-culture and *circBTG2* overexpression inhibited proliferation and invasion of glioma cells, whereas *circBTG2* knockdown promotes tumor growth in vivo. The effects of *RBP-J* OE Mφ-Exos on glioma cells can be reversed by the *circBTG2* knockdown. In conclusions, Exo-*circBTG2* secreted from *RBP-J* OE Mφ inhibits tumor progression through the *circBTG2*/miR-25-3p/*PTEN* pathway, and *circBTG2* is probably a diagnostic biomarker and potential target for glioma therapy.

## Introduction

Gliomas are primary malignancy commonly seen in the nervous system [1, 2], which are featured with heterogeneous genetic molecular aberrations [3]. Glioblastoma multiforme (GBM) is the most aggressive type with repeated relapse. The median survival rate of GBM patients is only 14.6 months even after standard advanced surgery and chemoradiotherapy with temozolomide [4, 5]. Thus, top priority should be given to the probation of the possible molecular mechanisms by which gliomas progress, so as to improve the therapy of gliomas, especially GBM.

Increasing evidence unveils that the exosomes (Exos) mediate the interactions between macrophages and cancer cells [6–8]. M2 Macrophage-derived exosomes (Mφ-Exos) were found to boost cancer cells to migrate and invade [9], and tumor-associated Mφ-Exos facilitate gastric cancer cells to migrate *via* transfer of functional Apolipoprotein E [10]. Besides, Mφ-Exo-miR-501-3p contributes to progression of PDAC *via* the TGF-β signaling pathway mediated by *TGFBR3* [11]. Downregulated lncRNA *SBF2-AS1* in M2 Mφ-Exos raises miR-122-5p to restrict XIAP, thus curbing PC development [12]). M2 bone marrow-derived Mφ-Exos elevate miR-21 to accelerate immune escape of gliomas *via* modulating *PEG3* [13].

Circular RNAs (circRNAs) have also been found in Exos [14, 15], and they are thought to modulate the expression of genes and miRNAs [16]. Exo-circRNAs can promote malignant phenotype of peripheral cells in cholangiocarcinoma [17]. Recent studies have unveiled the involvement of circRNAs in glioma progression by competitive sponging miRNAs [18, 19]. For instance, *circ_0037655* is able to boost gliomas to progress *via* controlling miR-214/PI3K signal transduction [20]. However, whether Mφ-Exo-circRNAs can regulate the progression of gliomas is unclear.

The Notch pathway is involved in several core cellular processes, such as proliferation and tumor development [21, 22], and it is also believed to be responsible for the activation and differentiation of macrophages [23–25]. The recombination signal-binding protein-Jκ (*RBP-J*) is a transcriptional regulator that is often used as a marker for the activation of Notch signaling [26]. Notch intracellular domains are released by Notch ligands and translocate to the nucleus where they bind to *RBP-J* [27]. Loss of the Notch effector *RBP-J* promotes tumorigenesis [28]. Moreover, Notch-*RBP-J* signal transduction regulates the transcription factor IRF8 to facilitate inflammatory macrophage polarization [29].

In the current research, we probed the impacts of *RBP-J* OE Mφ-Exos (exosomes derived from *RBP-J*-overexpressed macrophages) on glioma cell proliferation and invasion and compared them with Exos from THP-1-like macrophages (WT Mφ-Exos) [30]. To further understand the regulatory mechanism of *RBP-J* OE Mφ-Exos in gliomas, we also determined the differentially regulated circRNAs when *RBP-J* was upregulated in Mφ-Exos. In addition, we identified the miRNA binding partners of the circRNA and their targets. This study aimed at identifying pathways that are uniquely expressed in glioma progression to understand the mechanisms of Mφ-Exo-circRNAs, and determining diagnostic biomarkers and potential therapeutic targets.

## Results

### RBP-J is lowly expressed in the macrophages from the glioma tissues and related to the prognosis of glioma patients

**Fig. 1:**
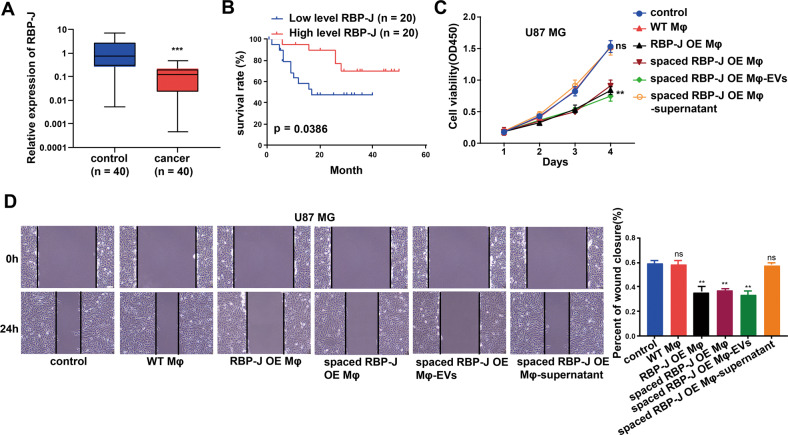
*RBP-J* OE Mφ inhibit proliferation and invasion of glioma cells through extracellular vesicles. **A** *RBP-J* expression in the microphages from glioma tissues and paired normal tissue samples (*n* = 40) using qRT-PCR. **B** Kaplan–Meier analysis of overall survival of glioma patients stratified by *RBP-J* expression. **C** Cell proliferation in glioma cell lines U87 MG co-cultured with WT Mφ, *RBP-J* OE Mφ, spaced *RBP-J* OE Mφ, spaced *RBP-J* OE Mφ-EVs or *RBP-J* OE Mφ-supernatant was assessed by CCK-8. **D** Wound healing assay is performed to detect cell migration (bar = 100 μm). All experiments were performed three times. **P < 0.01, ***P < 0.001 for statistical differences, EVs Extracellular vesicles, ns no significance.

Glioma tissues (*n* = 40) and para-tumor tissues (*n* = 40) were obtained from 40 patients. The expression of *RBP-J* in the macrophages from the glioma tissue samples and adjacent normal tissue samples was detected using qRT-PCR. Results showed that *RBP-J* is lowly expressed in the macrophages from the glioma tissues (Fig. 1A). The correlation between *RBP-J* expression in the macrophages of glioma tissue samples and clinicopathological parameters of glioma patients is shown in Table 1. The median expression level of *RBP-J* in the macrophages of glioma tissues was taken as the cut-off value for high and low levels. We found that higher *RBP-J* expression was associated with lower glioma Grade (*P* < 0.05), but there was no association between the expression level of *RBP-J* and other clinicopathological parameters in glioma such as age and gender (*P* > 0.05). In addition, the survival rate of glioma patients with highly expressed *RBP-J* in the macrophages of glioma tissues was better than that of patients with lower *RBP-J* expression in the macrophages of glioma tissues (Fig. 1B).

**Table 1:** Association between *RBP-J* expression in the macrophages from the glioma tissue samples and clinicopathological features of glioma.

Feathers	Number	High	Low	*P* value
All cases	40	20	20	
Age(years)				0.5273
≤48	21	12	9	
>48	19	8	11	
Gender				1.0000
Male	23	12	11	
Female	17	8	9	
Grade				**0.0115**
I-II	14	8	6	
III	16	11	5	
IV	10	1	9	

Total data of 40 glioma patients were analyzed. The expression of *RBP-J* in the macrophages from the glioma tissue samples was assayed by qRT-PCR, and the median expression level of *RBP-J* was used as the cutoff. Data were analyzed by Fisher’s exact test or Chi-square test. *P*-value in bold indicates statistically significant.

### RBP-J OE Mφ inhibit proliferation and invasion of glioma cells through extracellular vesicles

To further investigate the effects of the *RBP-J* OE Mφ on the proliferation and migration of glioma cells, we cocultured U87 MG cells with Mφ for 5 days and measured cell proliferation and migration with CCK-8 and wound healing assays. As demonstrated in Fig. 1C, D, *RBP-J* OE Mφ significantly inhibited cell proliferation and migration of U87 MG cells compared with WT Mφ. We wonder whether Mφ play a role through direct contact or indirect contact. Thus, we co-culture Mφ and U87 cells in separate spaces, allowing only the medium to contact each other. Then we found that *RBP-J* OE co-culture with Mφ could also significantly inhibited cell proliferation and migration of U87 MG cells (Fig. 1C, D). This means that Mφ play a role through indirect contact. Nevertheless, whether it is through extracellular vesicles or soluble small molecules still needs to be further explored. We extracted extracellular vesicles and supernatant of Mφ respectively. As shown in Fig. 1C, D, we found that Mφ play a role through extracellular vesicles.

### RBP-J OE Mφ-Exos inhibit proliferation and invasion of glioma cells

**Fig. 2:**
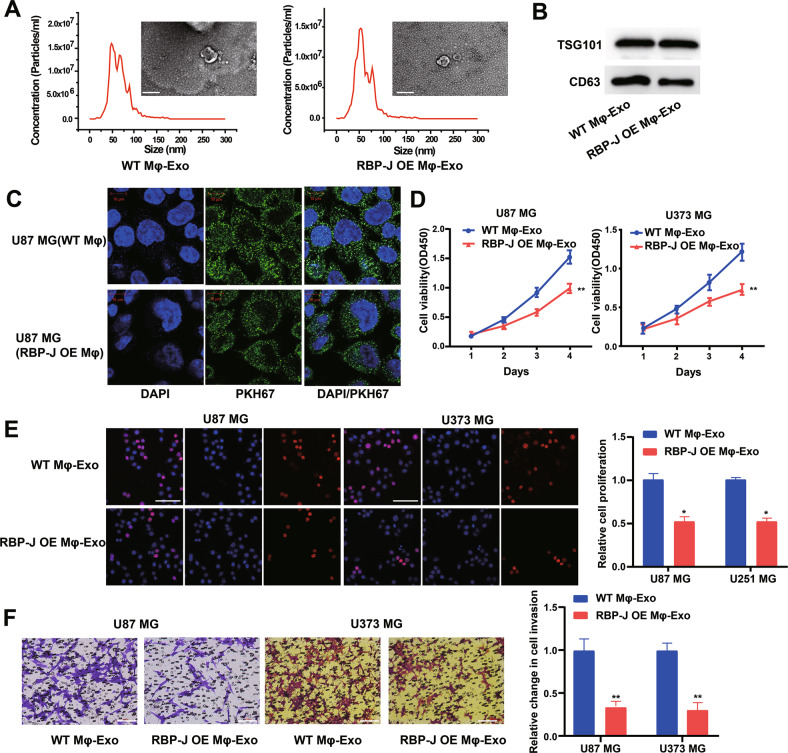
Exosomes derived from *RBP-J* overexpressed macrophages inhibit proliferation and invasion of glioma cells. **A** Exosomes isolated from WT THP-1 derived macrophages (WT Mφ-Exo) and *RBP-J*-overexpressed macrophages (*RBP-J* OE Mφ-Exo) imaged by transmission electron microscopy (TEM) and their size distribution were measured using NTA. Scale bar = 50 nm. **B** Levels of exosome markers CD63 and TSG101 in WT or *RBP-J* OE Mφ-Exo were determined by Western blotting. **C** WT Mφ-Exo or *RBP-J* OE Mφ-Exo were labeled with PKH67; green represents PKH67, and blue represents nuclear DNA staining by DAPI. U87 MG cells were incubated with WT Mφ-Exo or *RBP-J* OE Mφ-Exo for 3 h. **D**, **E** Cell proliferation in glioma cell lines U87 MG and U373 MG treated with WT Mφ-Exo or *RBP-J* OE Mφ-Exo was assessed by CCK-8 (**D**) and EdU assay (**E**). **F** Transwell invasion assay is performed to indicate cell invasion (bar = 100 μm). All experiments were performed three times. *P < 0.05 and **P < 0.01 for statistical differences.

WT Mφ-Exos and *RBP-J* OE Mφ-Exos were isolated by ultracentrifugation and characterized by TEM and NTA (Fig. 2A). To further confirm the identity of the Exos, the expression levels of CD63 and TSG101 (Exo markers) were evaluated. Western blot assessment showed that the isolated Exos were enriched with CD63 and TSG101 (Fig. 2B). These data indicate the successful isolation of Exos from WT Mφ-Exos and *RBP-J* OE Mφ-Exos. Then, exosomes derived from WT Mφ and *RBP-J* OE Mφ cells were labeled with a green fluorescent marker, PKH67. After recipient cells (U87 MG cells) were incubated with labeled WT Mφ-Exos and *RBP-J* OE Mφ-Exos for 3 h, PKH67 was localized in the cytoplasm of recipient cells (Fig. 2C).

To further investigate the effects of these two groups of Exos on the proliferation of glioma cells, we cocultured U87 MG or U373 MG cells with Exos for 5 days and measured cell proliferation through CCK-8 and EdU experiments. As demonstrated in Fig. 2D, E, the presence of *RBP-J* OE Mφ-Exos significantly curbed U87 MG and U373 MG cells to proliferate when compared with WT Mφ-Exos. In addition, Transwell invasion assays indicated that *RBP-J* OE Mφ-Exos were able to inhibit invasion of U87 MG and U373 MG cells (Fig. 2F). Overall, these results confirm that the overexpression of *RBP-J* in Exos can suppress glioma cells to proliferate and invade.

### Expression profiles of circRNAs in RBP-J OE Mφ-Exos

**Fig. 3:**
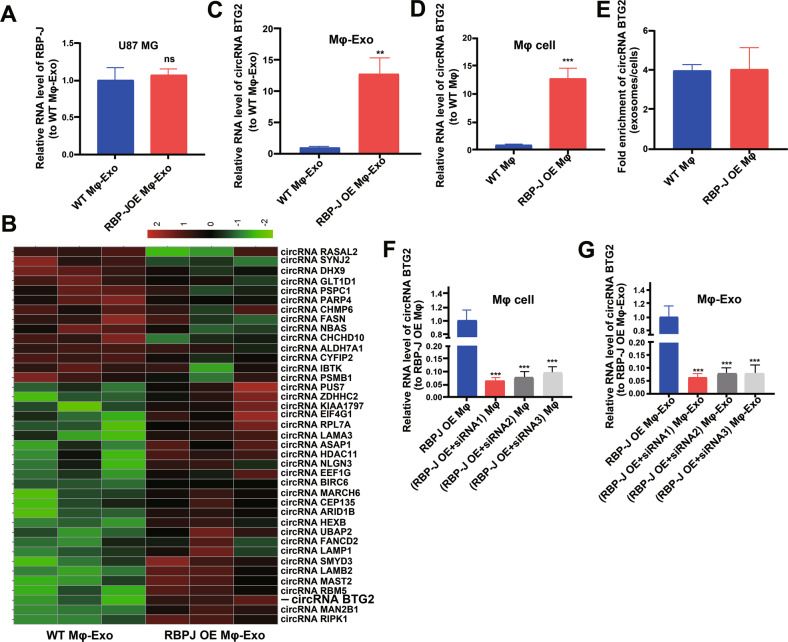
circRNA expression profiles in exosomes derived from *RBP-J*-overexpressed macrophages. **A** The relative expressions of *RBP-J* in WT Mφ-Exos treated U87 MG cells and *RBP-J* OE Mφ-Exos treated U87 MG cells were detected by qRT-PCR. **B** Cluster heatmap showing 39 aberrantly expressed circRNAs, including 25 upregulated and 14 downregulated circRNAs in exosomes derived from *RBP-J*-overexpressed macrophages compared to the controls. The red color represents high expression, whereas the green color represents low expression. **C**, **D** The relative expression of circRNA BTG2 in Mφ-Exo (**C**) and Mφ cells (**D**) was validated by qRT-PCR. **E** The fold change of circRNA BTG2 expression between the exosomes and their corresponding producer cells. **F**, **G** The qRT-PCR assay indicated the difference in the circRNA BTG2 expression in *RBP-J*-overexpressed Mφ cells transfected with or without si-circRNA (**F**), as well as in exosomes from those cells (**G**). **P < 0.01 and ***P < 0.001 for statistical differences, ns: no significance.

We wonder whether *RBP-J* OE Mφ-Exos could influence the expression of *RBP-J* in glioma cells. We performed qRT-PCR to detect the expression of *RBP-J* in the WT Mφ-Exos treated U87 MG cells, and *RBP-J* OE Mφ-Exos treated U87 MG cells. Results showed that there is no significant difference of *RBP-J* level between WT Mφ-Exos treated U87 MG cells and *RBP-J* OE Mφ-Exos treated U87 MG cells (Fig. 3A). This result means that *RBP-J* OE Mφ-Exos can’t influence the expression of *RBP-J* in U87 MG cells. Thus, we wonder whether there are different expressions of circRNAs between WT Mφ-Exos and *RBP-J* OE Mφ-Exos. The circRNA profiles in *RBP-J* OE Mφ-Exos and WT Mφ-Exos were evaluated using a circRNA microarray technique. 39 circRNAs were differentially expressed (*P* < 0.05 and log2FC > 2.0 or < −2.0) in *RBP-J* OE Mφ-Exos and the controls (Fig. 3B). Among them, 25 circRNAs dramatically rose up and 14 ones evidently declined. *circBTG2* with the most obvious rising trend was selected and validated to be present in *RBP-J* OE Mφ-Exos and WT Mφ-Exos by qRT-PCR (Fig. 3C). In the meantime, it was unveiled that *circBTG2* was expressed in the *RBP-J* OE Mφ at a notably higher level relative to that in the WT Mφ cells (Fig. 3D). Compared with those in the producer cells, the levels of *circBTG2* are enriched by approximately 4 folds in the *RBP-J* OE Mφ-Exos and WT Mφ-Exos (Fig. 3E). This result means that circBTG2 is enriched in exosomes. According to the circBase (http://www.circbase.org), *circBTG2* (chr1:203274663-203278729) was derived from *BTG2*.

### Mφ-Exo-circBTG2 inhibits glioma cells to proliferate and invade

**Fig. 4:**
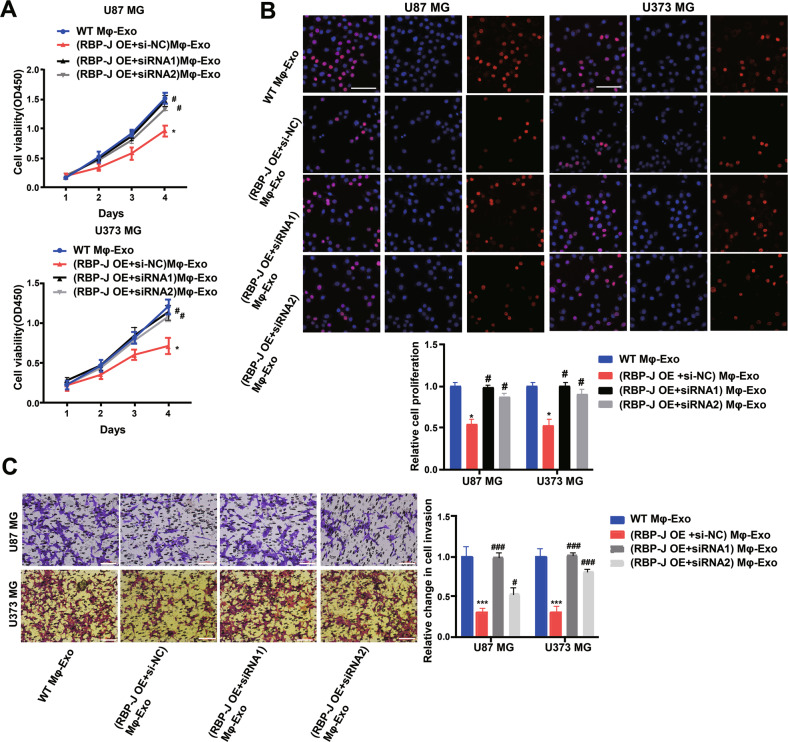
Mφ-Exo-circRNA BTG2 inhibits proliferation and invasion of glioma cells. To remove circRNA BTG2 from exosomes, siRNA of circRNA BTG2 was transfected into THP-1 cells and Mφ-Exo were collected at 48 h post-transfection (*RBP-J* OE Mφ-Exo-si-circRNA). Glioma cell lines U87 MG and U373 MG were cocultured with WT Mφ-Exo, *RBP-J* OE Mφ-Exo or (*RBP-J* OE + si-circRNA) Mφ-Exo. **A**, **B** Cell proliferation in glioma cell lines U87 MG and U373 MG was assessed by CCK-8 assay (**A**) and EdU assay (**B**). **C** Cell invasion in glioma cell lines U87 MG and U373 MG was assessed by Transwell assay (bar = 100 μm). All experiments were performed three times. *^#^*P* < 0.05 and ***^###^*P* < 0.001 as indicated. * vs. WT Mφ-Exo, # vs. *RBP-J* OE Mφ-Exo.

Since the *circRNA BTG2* level was the highest in *RBP-J* OE Mφ-Exos, to remove its expression from Exos, the siRNA of *circBTG2* was transfected into Mφ cells for 48 h, after which Exos were collected (Fig. 3F, G). Next, glioma cell proliferation and invasion were investigated by coculturing cells with Mφ-Exos. The inhibitory effects of *RBP-J* OE Mφ-Exos on the proliferation and invasion of glioma cells (U87 MG and U373 MG) were eliminated when *circBTG2* was knocked down in Mφ (Fig. 4A–C). This would be expected if there was an association between the expression of *RBP-J* and *circBTG2*.

To continuously figure out the biological role of *circBTG2* in glioma cells, U87 MG and U373 MG cells underwent transfection with a *circBTG2* overexpression vector (Supplementary Fig. S1A). The results unveiled that *circBTG2* overexpression significantly inhibited glioma cells to proliferate and invade (Supplementary Fig. S1B–D). As with the overexpression of *RBP-J*, the overexpression of *circBTG2* inhibited proliferation and invasion of glioma cells.

### circBTG2 acts as a sponge for miR-25-3p

**Fig. 5:**
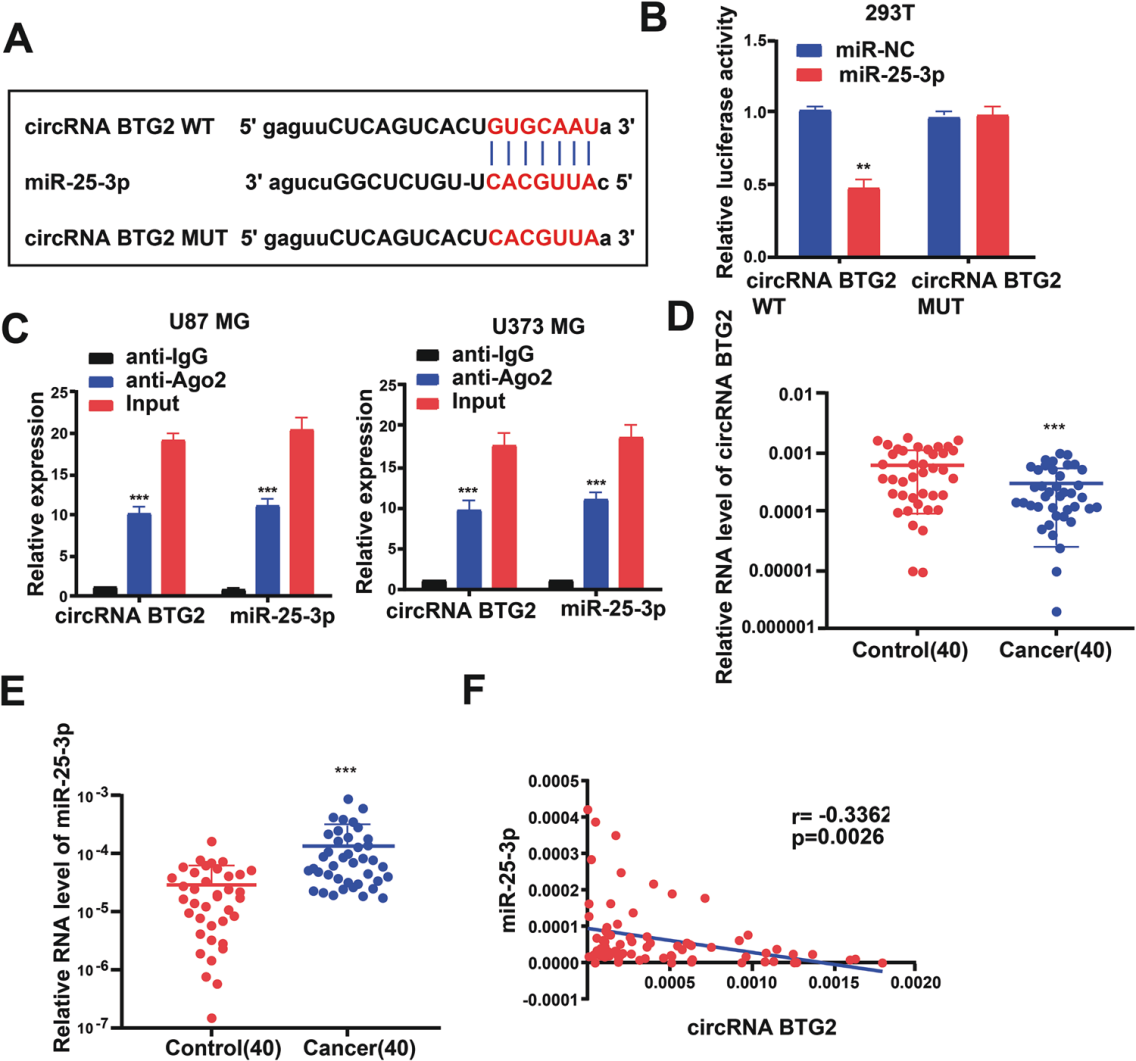
circRNA BTG2 acts as a sponge for miR-25-3p. **A** Putative complementary sites within miR-25-3p and circRNA BTG2 were predicted by bioinformatics analysis. **B** Dual-luciferase reporter assays demonstrate that miR-25-3p is a direct target of circRNA BTG2 in 293 T cells. **C** The Ago2 RIP showed that Ago2 significantly enriched circRNA BTG2 and miR-25-3p. **D** The expression level of circRNA BTG2 in 40 glioma tissues and matched para-carcinoma normal tissues was determined by qRT-PCR. **E** The expression level of miR-25-3p in the above tissues was determined by qRT-PCR. **F** The expression levels of miR-25-3p are negatively correlated with circRNA BTG2 in glioma tissues. **P < 0.01 and ***P < 0.001 for statistical differences.

For discovering more about the specific regulation of *circBTG2*, we performed bioinformatics prediction (starBase). Bioinformatics analysis predicted that *circBTG2* and miR-25-3p possessed complementary binding sites (Fig. 5A). Then we carried out a dual-luciferase experiment in 293 T cells to confirm this interaction by mutating the predicted binding site in *circBTG2*. It was unveiled that the luciferase activity was reduced only in presence of WT *circBTG2* and miR-25-3p mimics in 293 T cells (Fig. 5B), which was further validated using an Ago2 RIP assay. Ago2 significantly enriched RNA levels of both *circBTG2* and miR-25-3p (Fig. 5C). Besides, levels of *circBTG2* and miR-25-3p were also analyzed in glioma and matched para-carcinoma tissues, and the results further substantiated that glioma tissues exhibited a lower *circBTG2* level (Fig. 5D) and a higher miR-25-3p level (Fig. 5E). Besides, Pearson’s analysis confirmed a negative interrelation between *circBTG2* and miR-25-3p in glioma and matched para-carcinoma tissues (Fig. 5F). This indicates that *circBTG2* may compete to bind to miR-25-3p as a sponge and prevent it from regulating other pathways.

### circBTG2 represses glioma cells to proliferate and invade via the miR-25-3p/PTEN pathway

**Fig. 6:**
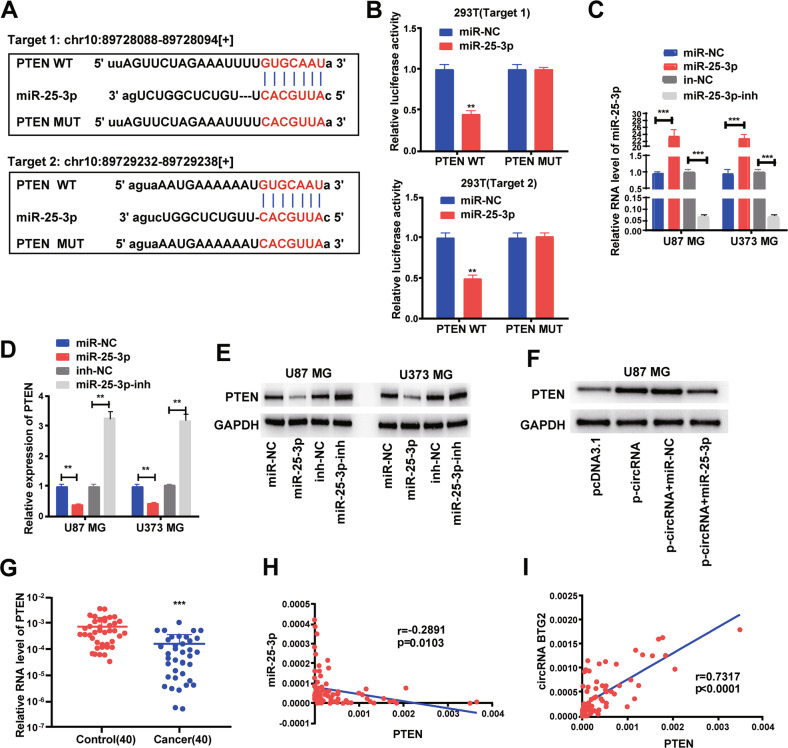
circRNA BTG2/ miR-25-3p axis is critical for PTEN expression. **A** Bioinformatics analysis revealed the predicted binding sites between PTEN and miR-25-3p. **B** Luciferase reporter assay demonstrated miR-25-3p mimics significantly decreased the luciferase activity of PTEN-WT in HEK293T cells. **C** The transfection efficiency of miR-25-3p mimics and inhibitor in U87 MG and U373 MG cells. **D**, **E** The mRNA (**D**) and protein (**E**) level of PTEN was detected through qRT-PCR and western blotting after transfection with miR-25-3p mimics and inhibitor in U87 MG and U373 MG cells. **F** circRNA BTG2 overexpression upregulated PTEN, this effect can be reversed by co-transfection with miR-25-3p mimics in U87 MG cells. **G** The expression levels of PTEN in 40 glioma tissues and matched para-carcinoma normal tissues was determined by qRT-PCR. **H** Expression levels of PTEN negatively correlated with miR-25-3p in glioma tissues. **I** Expression levels of PTEN positively correlated with circRNA BTG2 in glioma tissues. **P < 0.01 and ***P < 0.001 for statistical differences.

We next probed the potential binding sites of miR-25-3p. Target prediction and assessment were implemented using starBase (http://starbase.sysu.edu.cn) and miRDB (http://mirdb.org), which identified that miR-25-3p probably interacts with *PTEN*, a tumor suppressor gene implicated in several cancers [31, 32]. Later, we mutated two potential miR-25-3p target sites in *PTEN* (Fig. 6A) and performed a luciferase reporter experiment., which ascertained that miR-25-3p overexpression in HEK293T cells dramatically weakened the luciferase activity of *PTEN* at both target sites (Fig. 6B). Thereafter, we examined the transfection efficiency of miR-25-3p mimics and inhibitor (Fig. 6C). The mRNA and protein levels of *PTEN* dropped down in U87 MG and U373 MG cells transfected with miR-25-3p mimics but rose up in glioma cells undergoing miR-25-3p inhibitor transfection (Fig. 6D, E). Overexpression of *circBTG2* upregulated *PTEN* whereas miR-25-3p mimics transfection reversed it in U87 MG cells (Fig. 6F). Relative to the matched para-carcinoma tissues, *PTEN* was expressed at a lower level in glioma tissues (Fig. 6G). In glioma tissues, *PTEN* was negatively correlated with miR-25-3p, but positively correlated with *circBTG2* expression (Fig. 6H, I). Further, *circBTG2* overexpression inhibited cells to proliferate and invade whereas miR-25-3p mimics transfection reversed it in U87 MG and U373 MG cells (Supplementary Fig. S2A–C). It can be assumed that *circBTG2* inhibits proliferation and invasion in glioma cells by sponging miR-25-3p and upregulating *PTEN* expression.

### RBP-J OE Mφ-Exo inhibits tumor growth through the circBTG2/miR-25-3p/PTEN pathway in vivo

**Fig. 7:**
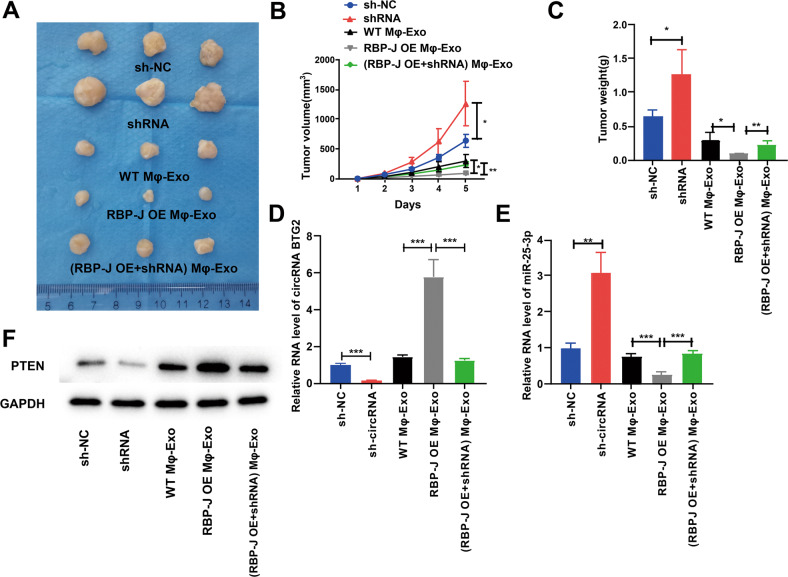
*RBP-J* OE Mφ-Exos inhibit tumor growth by circRNA BTG2/miR-25-3p/PTEN pathway in mouse xenograft tumor model. **A** Representative images of xenograft tumors (three mice per group) in nude mice. **B** Tumor volume is monitored every 7 days for 35 days. **C** The weights of xenograft tumors are summarized. **D** qRT-PCR verification of the expression of circRNA BTG2 in tumors (*n* = 3). **E** qRT-PCR verification of the expression of miR-25-3p in tumors (*n* = 3). **F** The protein expression of PTEN was detected by western blotting in tumors (*n* = 3). *P < 0.05, **P < 0.01, and ***P < 0.001 for statistical differences.

For proving the effect of Mφ-Exo-*circBTG2* on the modulation of glioma growth in vivo, U87 MG cells undergoing transfection with sh-circRNA or sh-NC or coculture with WT Mφ-Exos, *RBP-J* OE Mφ-Exos or (*RBP-J* OE + sh-circRNA) Mφ-Exo were subcutaneously injected into nude mice. Tumors cultured with *RBP-J* OE Mφ-Exos were significantly smaller, whereas those undergoing sh-circRNA transfection were significantly larger. The greatest differences in the tumor volume and weight were observed in the tumors between *RBP-J* OE Mφ-Exo group and sh-circRNA group (Fig. 7A–C). What’s more, the inhibitory effects of *RBP-J* OE Mφ-Exos on the tumor growth in vivo were eliminated when circRNA *BTG2* was knocked down in Mφ (Fig. 7A–C). The relative expression of *circBTG2* was the highest in the *RBP-J* OE Mφ-Exo group and the lowest in sh-circRNA group (Fig. 7D). While the relative expression of miR-25-3p was the lowest in the *RBP-J* OE Mφ-Exo group and the highest in sh-circRNA group (Fig. 7E). Meanwhile, protein levels of PTEN were the highest in *RBP-J* OE Mφ-Exo group and lowest in sh-circRNA group (Fig. 7F). These results signify that *RBP-J* OE Mφ-Exos might inhibit tumor growth through a *circBTG2*/miR-25-3p/*PTEN* pathway in xenograft tumor models.

## Discussion

Macrophages are abundant in the glioma tumoral environment and associated with chronic inflammation [33, 34]. Moreover, the macrophage environment is heterogenous with the progression of tumors dependent on alternatively polarized M2 macrophages and tumorigenic immune responses dependent on M1-polarized macrophages [35, 36]. Therefore, improving the understanding of macrophage regulation in the tumoral environment is important in developing effective therapies for gliomas. Notch-*RBP-J* signaling is believed to regulate TLR-induced inflammatory macrophage polarization by the indirect regulation of M1-specific genes [29].

In this study, we examined whether *RBP-J* overexpression in macrophages would influence glioma cells. We found that *RBP-J* OE Mφ-Exos could curb glioma cells to proliferate and invade. Furthermore, we probed their interrelations by investigating the differentially regulated circRNAs in Mφ-Exos with upregulated *RBP-J*. Using the circRNA microarray technique, we discovered that 39 Exo-circRNAs were differentially regulated in WT Mφ-Exos with *RBP-J* overexpression, of which 25 were upregulated and 14 were downregulated. Later, we selected the Exos with the highest *circBTG2* expression for further analysis. Then we unveiled that the inhibitory effects of *RBP-J* OE Mφ-Exos on the proliferation and invasion of glioma cells (U87 MG and U373 MG) were eliminated when *circBTG2* was knocked down in Exos. Meanwhile, *circBTG2* overexpression dramatically repressed glioma cells to proliferate and invade. These associations required further investigation, so we searched for miRNAs that may interact with *circBTG2*.

The public database (starBase) predicted that *circBTG2* may interact with miR-25-3p, which was validated *via* luciferase or RIP assays. Our studies proved that the overexpression of *circBTG2* could reduce miR-25-3p level. Then, a negative correlation between miR-25-3p and *circBTG2* in glioma and matched para-carcinoma tissues was confirmed by Pearson’s analysis. Thus, we deduced that *circBTG2* may repress miR-25-3p to prevent it from interacting in other pathways. StarBase revealed that miR-25-3p interacted with *PTEN*, a well-known tumor suppressor gene [31, 32]. Mutation or deletion of *PTEN via* complete loss of its locus on chromosome 10q is found in a multifold of GBMs [37, 38] and correlated with poor prognosis in diverse glioma subtypes [39, 40]. Furthermore, *PTEN* loss dramatically enhances gliomagenesis in quantities of murine model systems [41–43]. In the current research, we found that *circBTG2* overexpression upregulated *PTEN* and inhibited cells to proliferate and invade whereas miR-25-3p mimics transfection reversed them in glioma cells. What’s more, *PTEN* expression displayed a negative interrelation with miR-25-3p level and a positive correlation with *circBTG2* level in glioma tissues. In vivo assays further verified that *RBP-J* OE Mφ-Exos might inhibit tumor growth through a *circBTG2*/miR-25-3p/*PTEN* pathway in xenograft tumor models.

**Fig. 8:**
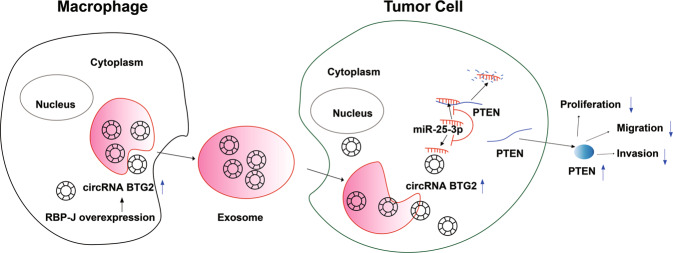
Graphpical Abstract of *RBP-J* OE Mφ-Exos-mediated glioma progression. RBP-J OE Mφ-Exo enhance circRNA BTG2 expression of glioma cells. Exosomal circRNA BTG2 may suppress the progression of glioma by acting as a ceRNA to competitively bind to miR-25-3p and regulate PTEN expression.

To conclude, *circBRG2* suppress glioma cells to proliferate and invade *via* the miR-25-3p/*PTEN* pathway. Moreover, *RBP-J* OE Mφ-Exo inhibits tumor growth by stimulating the *circBRG2*/miR-25-3p/*PTEN* pathway in vitro and in vivo (Fig. 8). The above results indicate that *RBP-J* OE Mφ-Exos probably play a potential regulation role in the glioma progression and *circBTG2* could be a biomarker for glioma diagnosis and potential target for glioma therapy.

## Materials and methods

### Cell culture and clinical specimens

Human monocytic cell line THP-1 and glioma cell lines (U87 MG and U373 MG) were purchased from ATCC and maintained according to ATCC guidelines. THP-1 cells were cultured in RPMI-1640 medium provided by Gibco (Shanghai, China), and glioma cells were cultured in Dulbecco’s Modified Eagle medium (DMEM, Gibco, China) with 10% heat-inactivated fetal bovine serum (FBS) from Thermo Fisher Scientific (Shanghai, China), 100 U/mL penicillin, and 100 µg/mL streptomycin from HyClone Laboratories (Beijing, China) at 37 °C in a moist incubator with 5% CO_2_ and used in the exponential growth phase.

Forty paired glioma tissues and para-tumor tissues were obtained from patients receiving surgery at The First people’s Hospital of Yancheng between 2015 and 2018. They were diagnosed by histopathology and received no treatment prior to the operation. Besides, all participants signed informed consent in written form before the research. This research gained the approval of the Ethics Committees of The First people’s Hospital of Yancheng and was conducted *as per* the Helsinki Declaration.

### Macrophage extraction

The tissue single cell suspension (2*10^8^/mL) was obtained and mixed into the separation solution (Sangon Biotech, Shanghai, China). The mixture was centrifuged for 20 min (1500 r/min) and the milky white macrophage layer was then collected. The macrophage layer was added into a test tube containing 5 mL of cell washing solution. After mixing, the macrophage layer was centrifuged (1800 r/min) and washed twice.

### Isolation of Exos derived from THP-1 Mφ cells with or without the overexpression of RBP-J

To obtain WT Mφ and *RBP-J* OE Mφ, THP-1 cells underwent transfection with the pCMV6 empty vector or pCMV6 overexpressing *RBP-J* (OriGene, Rockville, MD, USA) and seeded at 1 × 10^6^ cells/well in a six-well culture plate. Cellular debris were discarded from the collected culture medium that was centrifuged at 3000 × *g* for 15 min. Then Exoquick exosome precipitation solution (System Biosciences, CA, U.S.A.) was utilized for exosome separation [44–46].

### Nanoparticle tracking analysis (NTA)

We measured the exosome particle size and concentration using NTA with ZetaView PMX 110 (Particle Metrix, Meerbusch, Germany) and corresponding software ZetaView 8.04.02. Isolated exosome samples were appropriately diluted using 1X PBS buffer (Biological Industries, Israel) to measure the particle size and concentration. NTA measurement was recorded and analyzed at 11 positions. The ZetaView system was calibrated using 110 nm polystyrene particles. The temperature was maintained at around 23 °C and 37 °C.

### Transmission electron microscopy assay

Exos for transmission electron microscopy (TEM) were prepared as mentioned above.32 Briefly, Exos were first fixed in 2.5% glutaraldehyde (pH 7.2) at 4 °C, then washed in PBS, embedded in 10% gelatin and fixed in 1% osmium tetroxide for 60 min at indoor temperature. Next, the embedded Exos were cut into 1 mm-thick blocks and dehydrated with gradient alcohol. The alcohol was then replaced with gradient mixture of Quetol-812 epoxy resin and propylene oxide. Afterward, samples were embedded in Quetol-812 epoxy resin, polymerized at a temperature gradient, and cut into ultrathin sections using a Leica UC6 ultramicrotome. Finally, subsequent to dying by uranyl acetate and lead citrate, a transmission electron microscope was utilized for section observation.

#### Exosome labeling

Exosomes from 1.5 × 10^6^ cells were suspended in 100 μl of PBS with 1 ml of mixed PKH67 (Sigma, in Diluent C). After 4 min of incubation at room temperature, 2 ml of 0.5% bovine serum albumin (BSA) was added to terminate exosome labeling, and dyed exosomes were isolated using Exoquick exosome precipitation solution. Exosomes were suspended in 9.6 ml of basal medium, and 250 μl was added to the sub-confluent layer of U87 MG cells. After incubation for 3 h at 37 °C, cells were washed and fixed at room temperature. To stain the nuclei, 4′,6-diamidino-2-phenylindole (DAPI, Sigma) was added for 10 min, and the stained cells were observed with a fluorescence microscope (Zeiss, LSM700B, Germany).

### Microarray analysis

The isolation and quantification of the total RNAs were independently implemented using Trizol reagent provided by Life Technologies (Shanghai, China) and NanoDrop ND-1000. To enrich circRNAs and remove linear RNAs, RNase R provided by Epicenter Biotechnologies (Beijing, China) was utilized for RNA digestion. The enriched circRNAs were then amplified and labeled fluorescently using a Super RNA Labeling kit from Arraystar (Shanghai, China) *as per* the guideline of the manufacturer. In the meantime, we hybridize the labeled cRNAs onto an Arraystar Human circRNA Array V2 (8 × 15 K). Subsequent to rinsing and scanning of slides using an Agilent Scanner G2505C, the obtained images were assessed *via* Agilent Feature Extraction software (version11.0.1.1). Later, the limma package in R was utilized for quantile normalization and data assessment. Ultimately, fold change filtering and hierarchical clustering were used to determine circRNAs with differential expressions and their expression patterns.

### RNA extraction and quantitative real-time PCR (qRT-PCR)

The reverse transcription of mRNAs and circRNAs into cDNAs was implemented using a reverse transcription kit from Takara (Beijing, China), which were synthesized using the miRNA 1st-strand cDNA synthesis kit (Sangon Biotech, China). Next, cDNAs were subjected to RT-PCR on a Quantstudio™ DX system (Applied Biosystems, Singapore) under the following conditions: denaturation at 95 °C for 30 s and (denaturation at 95 °C for 5 s, at 60 °C for 10 s and at 72 °C for 30 s) × 40 cycles. Afterward, we utilized 2^-ΔΔCT^ to quantify mRNAs and circRNAs by normalizing to GAPDH [47] and to determine the relative expression subsequent to the normalization of miRNA expression to small nuclear U6. Each experiment was separately performed in triplicate. All PCR primers were listed in Table 2.

**Table 2:** Sequences of primers for qRT-PCR and siRNA related sequence.

Name		Sequence
circRNA BTG2	Forward	5’- TGGAAGAATGTACAGCTTATGGA-3’
	Reverse	5’- CTCGGGCTCAGTGAGAGGT-3’
PTEN	Forward	5’- TGGATTCGACTTAGACTTGACCT-3’
	Reverse	5’- GGTGGGTTATGGTCTTCAAAAGG-3’
GAPDH	Forward	5’-GGCTGTTGTCATACTTCTCATGG-3’
	Reverse	5’-GGATCTCGCTCCTGGAAGATG-3’
U6	Forward	5’-CTCGCTTCGGCAGCACA-3’
	Reverse	5’-AACGCTTCACGAATTTGCGT-3’
miR-25-3p	Forward	5’- ACACTCCAGCTGGGCAUUGCACUUGUCUCG-3’
	Reverse	5’- CTCAACTGGTGTCGTGGAGTCGGCAATTCAGTTGAGCGAGACAA-3’
circRNA BTG2 siRNA	Forward	5’- GUAGGAUAACAGGGUAACGCUUU-3’
	Reverse	5’- AGCGUUACCCUGUUAUCCUACUU-3’
miR-25-3p mimics	Sense	5’- CAUUGCACUUGUCUCGGUCUGA-3’
	Antisense	5’- AGACCGAGACAAGUGCAAUGUU-3’
miR-25-3p inhibitor	Sense	5’- UCAGACCGAGACAAGUGCAAUG-3’

### Cell transfection

CircRNA BTG anti-proliferation factor 2 (*BTG2*) (*circBTG2*) overexpression plasmid (p-circRNA) and its mimic pcDNA3.1, small interfering RNAs (siRNAs) targeting circRNAs and nonspecific negative control oligos (si-NC), miR-25-3p mimics, inhibitor and the negative control (NC), and the lentivirus targeting *circBTG2* were bought from GeneChem (Shanghai, China). Detailed sequences were depicted in Table 2. U87 MG and U373 MG cell lines underwent inoculation in six-well plates at 24 h prior to transfection with pcDNA3.1, p-circRNA, si-NC, si-circRNA, and miR-25-3p mimics or inhibitor under 50–60% cell confluence using Lipofectamine 3000 (Invitrogen) *as per* the guideline of the manufacturer. Later, the effects of knockdown or overexpression were examined by qRT-PCR using the RNAs that were extracted after 48-h transfection. For Exo treatment, glioma cells were cultured in medium containing 5 μg/ml Exos from WT Mφ, *RBP-J* OE Mφ or (si-circRNA + *RBP-J* OE) Mφ.

### Cell proliferation assays

Approximately 1.0 × 10^3^ transfected U87 MG and U373 MG cells were cultured in 96-well plates, and then underwent 1 h incubation with CCK-8 reagent (Beyotime, Shanghai, China). The absorbance at 450 nm was recorded using an Infinite M200 multimode microplate reader (Tecan, Shanghai, China).

After approximately 48 h transfection, the 5-ethynyl-2’-deoxyuridine (EdU) assay kit provided by Ribo (Guangzhou, China) was utilized to examine the proliferation of U87 MG and U373 MG cells. Specifically, cells were grown in culture medium containing EdU (Invitrogen) solution (1:1000). At the proliferative stage, the cells were labeled with EdU for 2 h, followed by rinsing with PBS (0.5 g/mL) thrice. Subsequently, the cells were stained by 4′,6-diamidino-2-phenylindole (DAPI) from Invitrogen for 10 min at indoor temperature in the dark and underwent PBS rinsing more than twice. Ultimately, assessment of the stained cells was implemented *via* the FACSCalibur DxP flow cytometer (BD Biosciences, Shanghai, China).

### Cell invasion assays

For invasion assays, the lower chambers were precoated with 100 μL of Matrigel (BD Bioscience, San Jose, CA, USA) for 30 min before the addition of medium to the chambers. The glioma cells (2 × 10^5^ cells/mL) were resuspended in DMEM medium. The upper chamber contained 100 μL of cell suspension medium, and 600 μL of complete medium was added to the bottom chamber. After incubating at 37 °C with 5% CO^2^ for 24 h, cells were fixed with 4% paraformaldehyde and stained with 0.1% crystal violet solution. The cells that passed through the filter were photographed and counted by inverted fluorescence microscopy (Leica Microsystems GmbH, Wetzlar, Germany) in four randomly selected fields.

### Luciferase reporter assay

Sequences of WT or MUT *circBTG2* or the full length of the 3′-UTR of *PTEN* with WT or MUT putative binding sites were interposed into the pmir-GLO vector from Promega Corp. (Beijing, China). 293 T cells seeded into 24-well plates underwent co-transfection with 50 nM miR-25-3p mimics or a NC and 80 ng WT or MUT plasmids using Lipofectamine 2000 (Invitrogen)and the 80 ng of plasmids were later added with 5 ng of pRL-SV40. Lastly, luciferase intensity was determined using the Dual-Luciferase Reporter Assay Kit from Promega (Beijing, China) and a microplate reader.

### RNA binding protein immunoprecipitation (RIP) assay

We carried out the RIP assay using a Magna RIP Kit from Millipore (Hongkong, China) as per the guideline of the manufacturer. Specifically, cells (2 × 10^7^) were lysed with the lysis buffer provided in the kit and the lysate was separately put into two tubes [one with anti-Argonaute2 (AGO2) antibody and the other with a nonspecific anti-IgG antibody (Millipore)]. The cell lysates were incubated nightlong at 4 °C, and then incubated with magnetic beads for a further hour. Proteinase K was then added for sample incubation at 55 °C for another hour. In the end, RNA extraction reagent (Solarbio, Beijing, China) was used to obtain the RNAs, and specific genes were detected and measured using qRT-PCR.

### Western blot analysis

Cell lysis was performed in RIPA buffer (Beyotime, Nantong, China) containing protease and phosphatase inhibitors (Beyotime). A BCA Protein Assay kit (Beyotime) was utilized to identify protein concentration, and the samples (40 µg proteins per lane) underwent SDS-PAGE with 10% gel for separation. Next, proteins were electrotransferred onto a PVDF membrane (Beyotime) that was sealed by 5% BSA (Beyotime) for 1 h at indoor temperature. Later, we incubated the membrane with primary antibodies against TSG101 (1:1000, ab125011, Abcam, Shanghai, China), CD63 (1:1000, ab217345, Abcam, Shanghai, China), *PTEN* (1:1000, ab267787, Abcam, Shanghai, China) and GAPDH (1:1000, ab8245, Abcam, Shanghai, China) at 4 °C nightlong. The membranes were then probed with HRP-labeled secondary antibodies (Beyotime, Nantong, China) at indoor temperature for 1 h and signals were detected by chemiluminescence.

### Xenograft nude mouse model

6–8-week-old adult male BALB/C nude mice (*n* = 3/group) were commercially provided by Shanghai SLAC Laboratory Animal Co., Ltd. (Shanghai, China) and reserved in a SPF environment with a LD (12: 12) cycle. All animal studies obtained the approval of the Institutional Animal Care and Use Committee of The First people’s Hospital of Yancheng and implemented in line with institutional and national guidelines. U87 MG cells undergoing stable sh-NC or sh-circRNA transfection, or WT Mφ-Exo, *RBP-J* OE Mφ-Exo or *RBP-J* OE Mφ-Exo-sh-circRNA (5 μg/ml) treatment were hypodermically injected into the nude mice (1 × 10^6^ cells per mouse) on the right upper back. Later, we utilized a caliper to determine the growth of tumor every 7 days for 35 days, and calculate its volume based on the formula: volume = (length × width^2^) /2. Five weeks later, we intraperitoneally injected overdose pentobarbital (>120 mg/kg body weight) to kill all the mice so that they were unable to spontaneously breath. Afterward, the xenograft tumor tissues were sampled for subsequent analyses.

#### Statistical assessment

GraphPad Prism 6.0 software provided by GraphPad Inc. (San Diego, CA, USA) was utilized to statistically evaluate data. Experimental results were presented as mean ± standard deviation (SD). The statistically significant differences between tumor tissues and para-tumor tissues were determined using paired Student’s *t*-test. Besides, the statistically significant differences between other two groups were detected using Mann–Whitney *U*-test or unpaired Student’s *t*-test in light of conditions. Furthermore, the comparisons among different groups (multigroup comparisons) were implemented by one-way ANOVA and the post hoc Bonferroni test. Lastly, Pearson’s correlation coefficient was determined to test associations among *circBTG2*, miR-25-3p, and *PTEN*. Log-rank test and Kaplan–Meier method were used to assess survival rates. Data concerning the association of *RBP-J* expression with clinicopathological features of glioma were analyzed by Fisher’s exact test. *P* < 0.05 signified statistically significant differences.

## Supplementary information

Original western blots

Supplemental material

Related manuscript file

## Data Availability

The data that support the findings of this study are available from the corresponding author upon reasonable request.
