# Supplementary material for: Exosomal circRNA BTG2 derived from RBP-J overexpressed-macrophages inhibits glioma progression via miR-25-3p/PTEN
Source: Cell Death Dis. 2022 May 28;13(5):506. doi: 10.1038/s41419-022-04908-4 (PMC9148311; doi:10.1038/s41419-022-04908-4)
Supplement: Supplementary file 1 — Original western blots [file 41419_2022_4908_MOESM1_ESM.docx]

Fig.2B-CD63


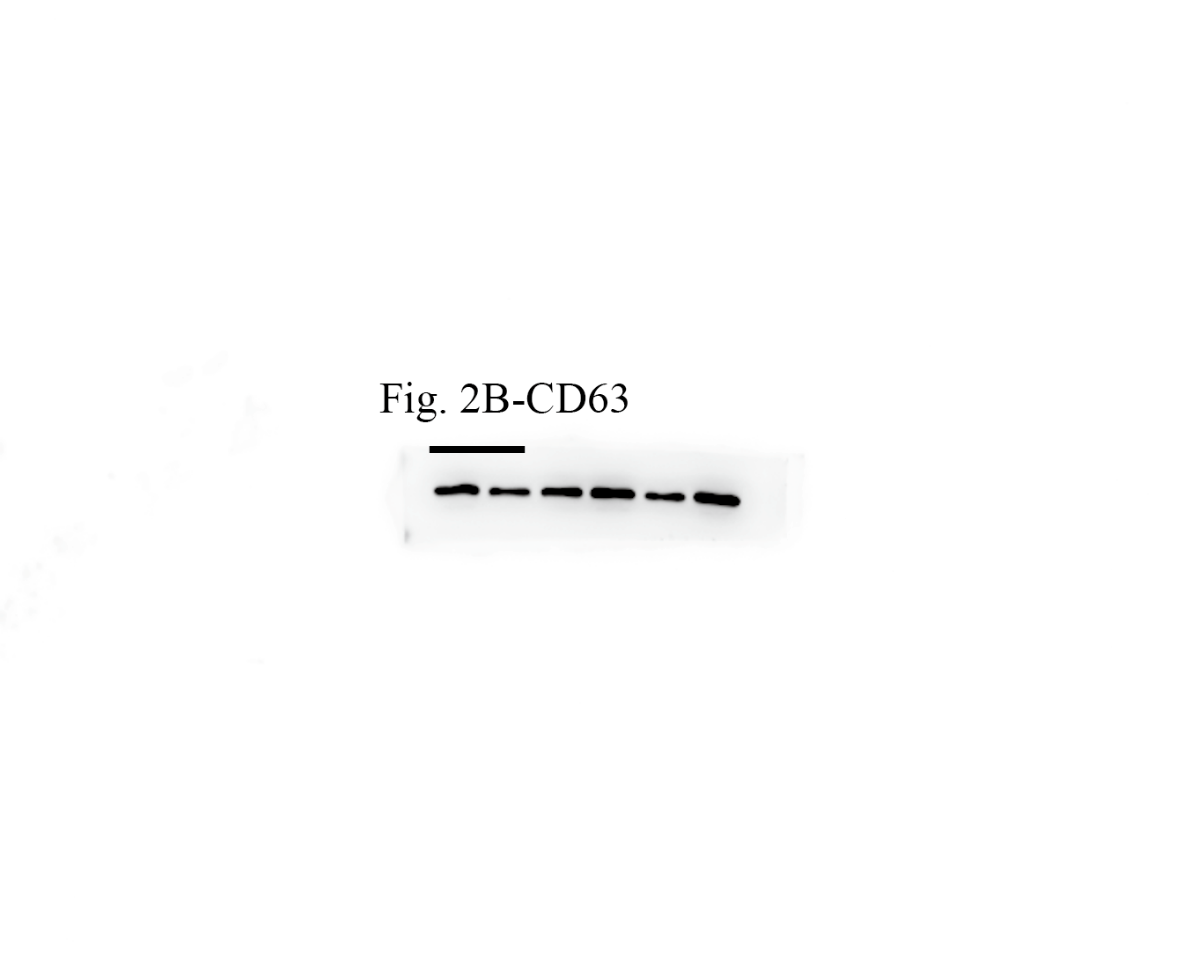


Fig.2B-CD63-marked





Fig.2B-TSG101


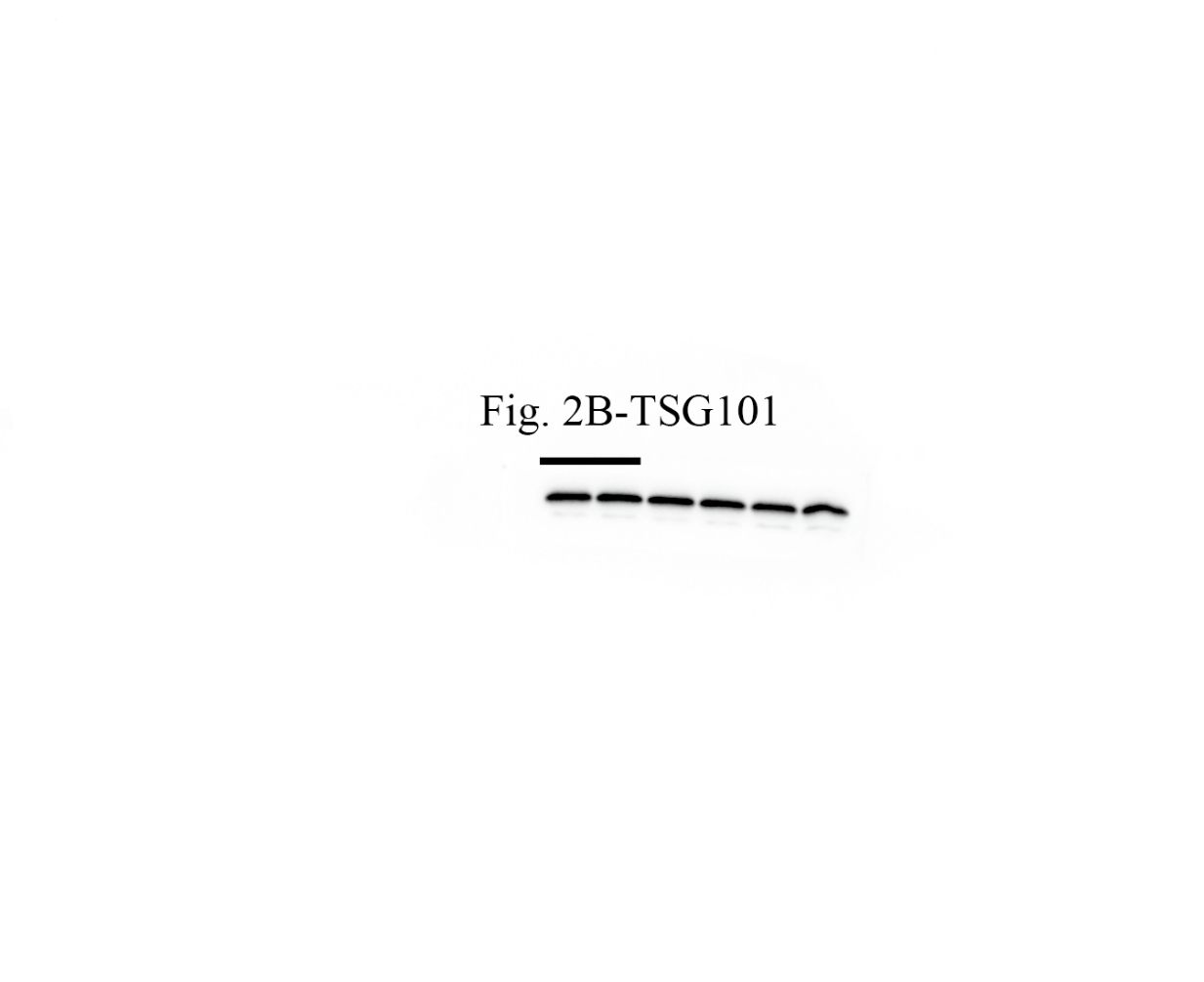


Fig.2B-TSG101-marked


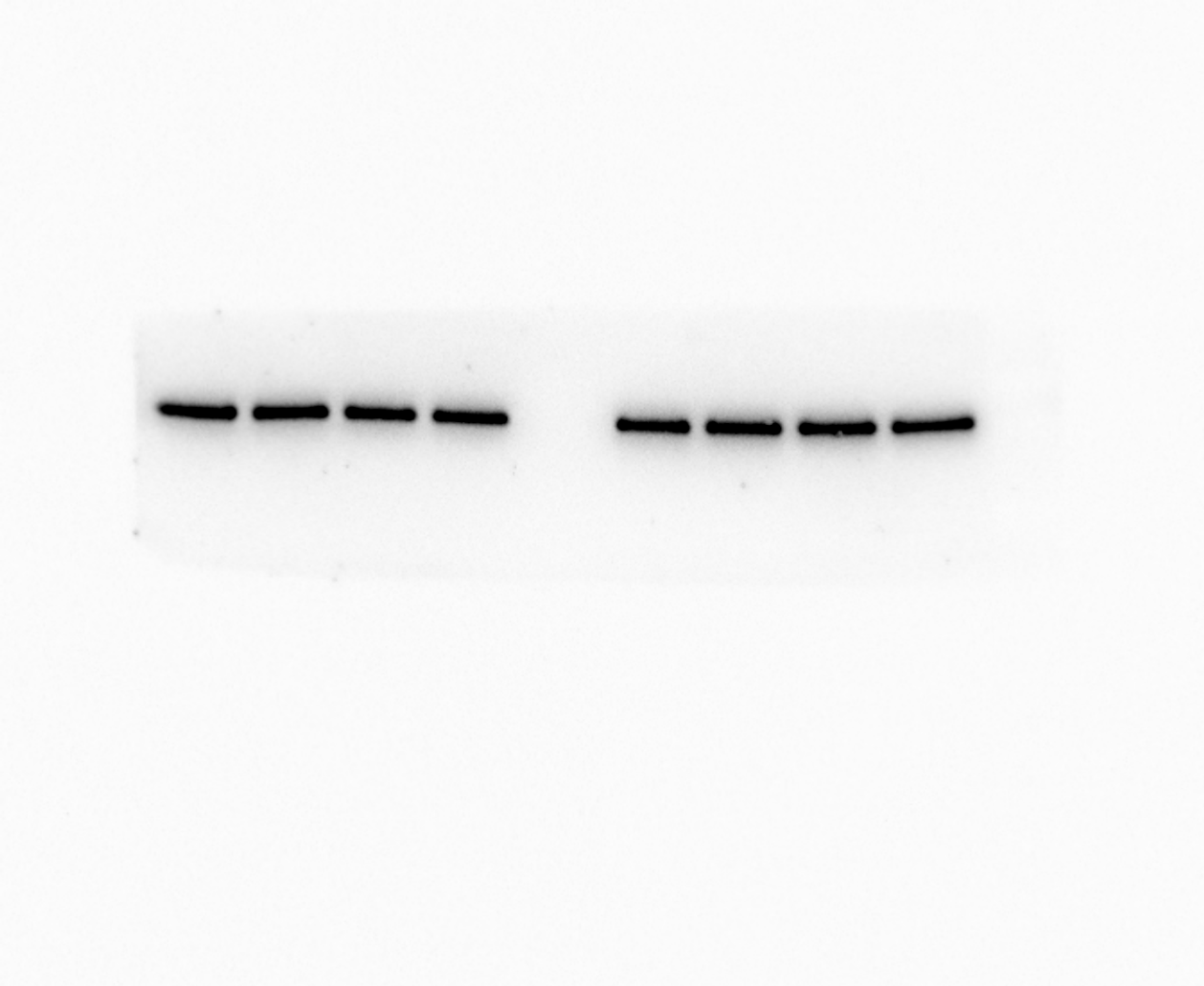


Fig.6E-GAPDH


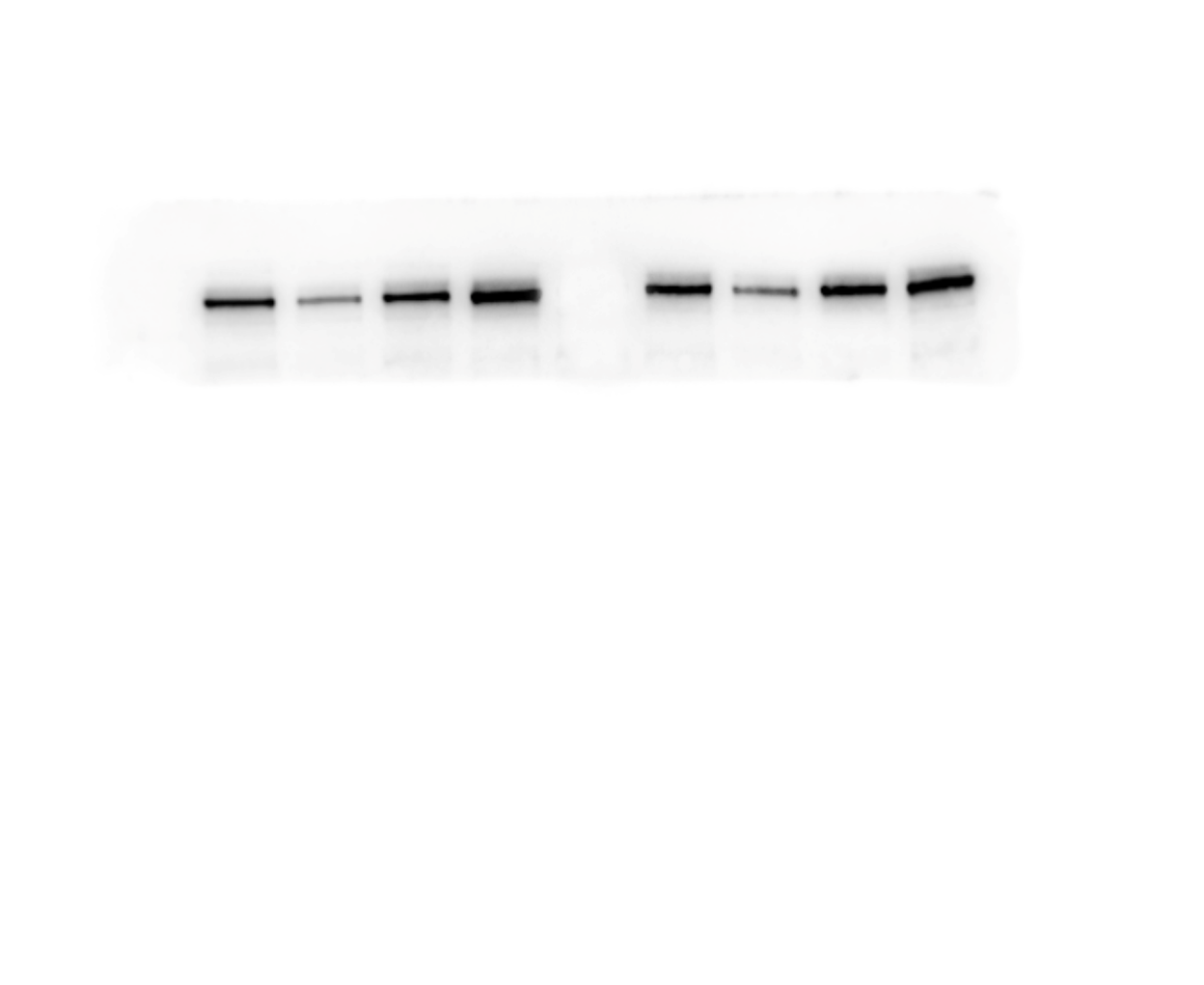


Fig.6E-PTEN


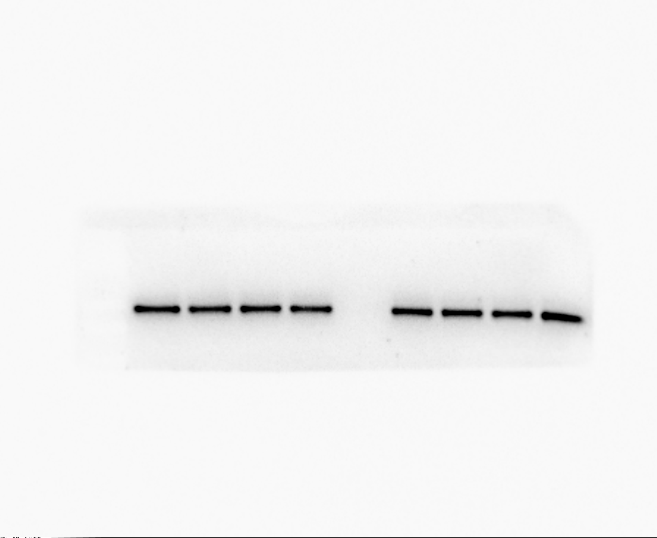


Fig.6F-GAPDH


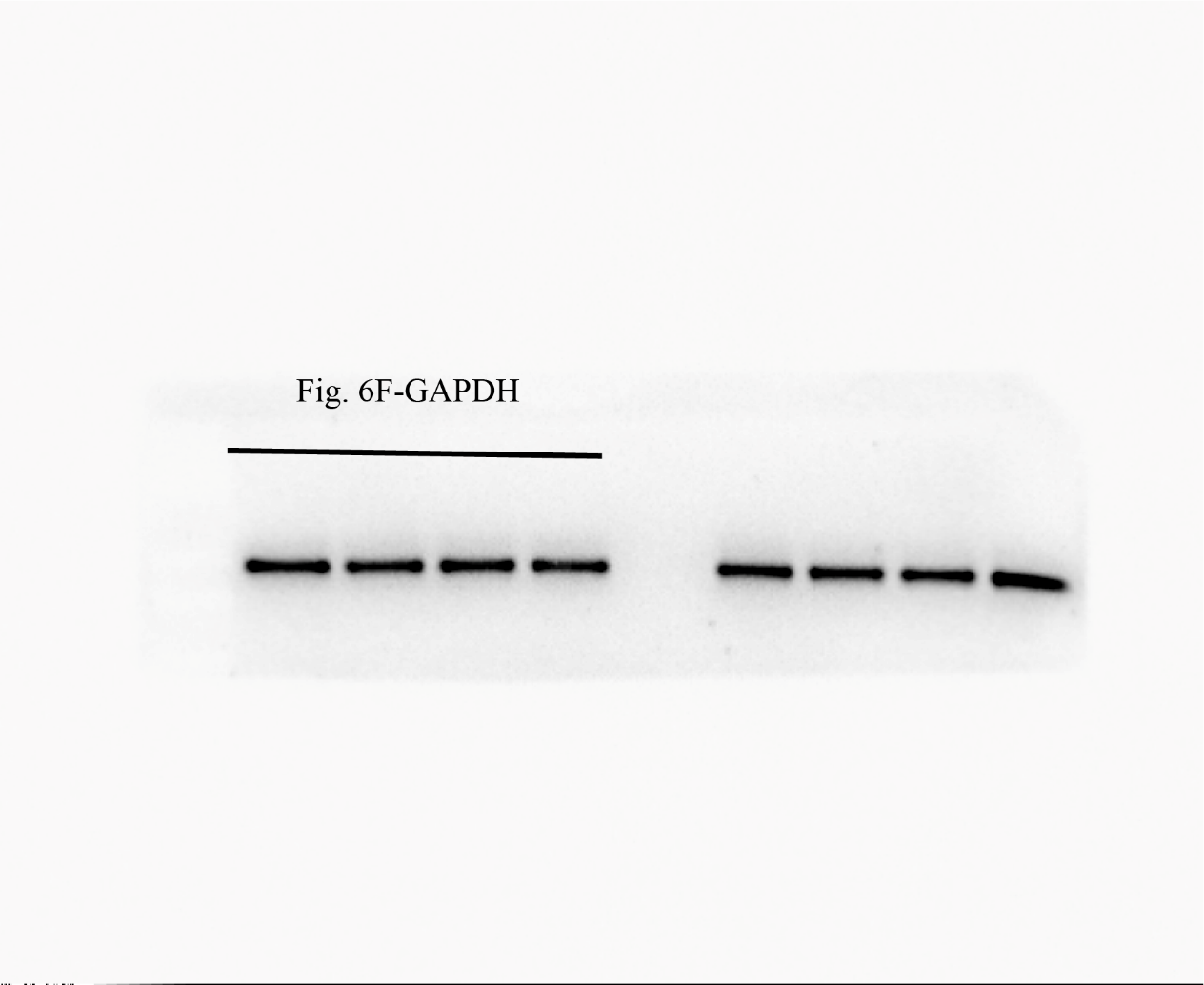


Fig.6F-GAPDH-marked


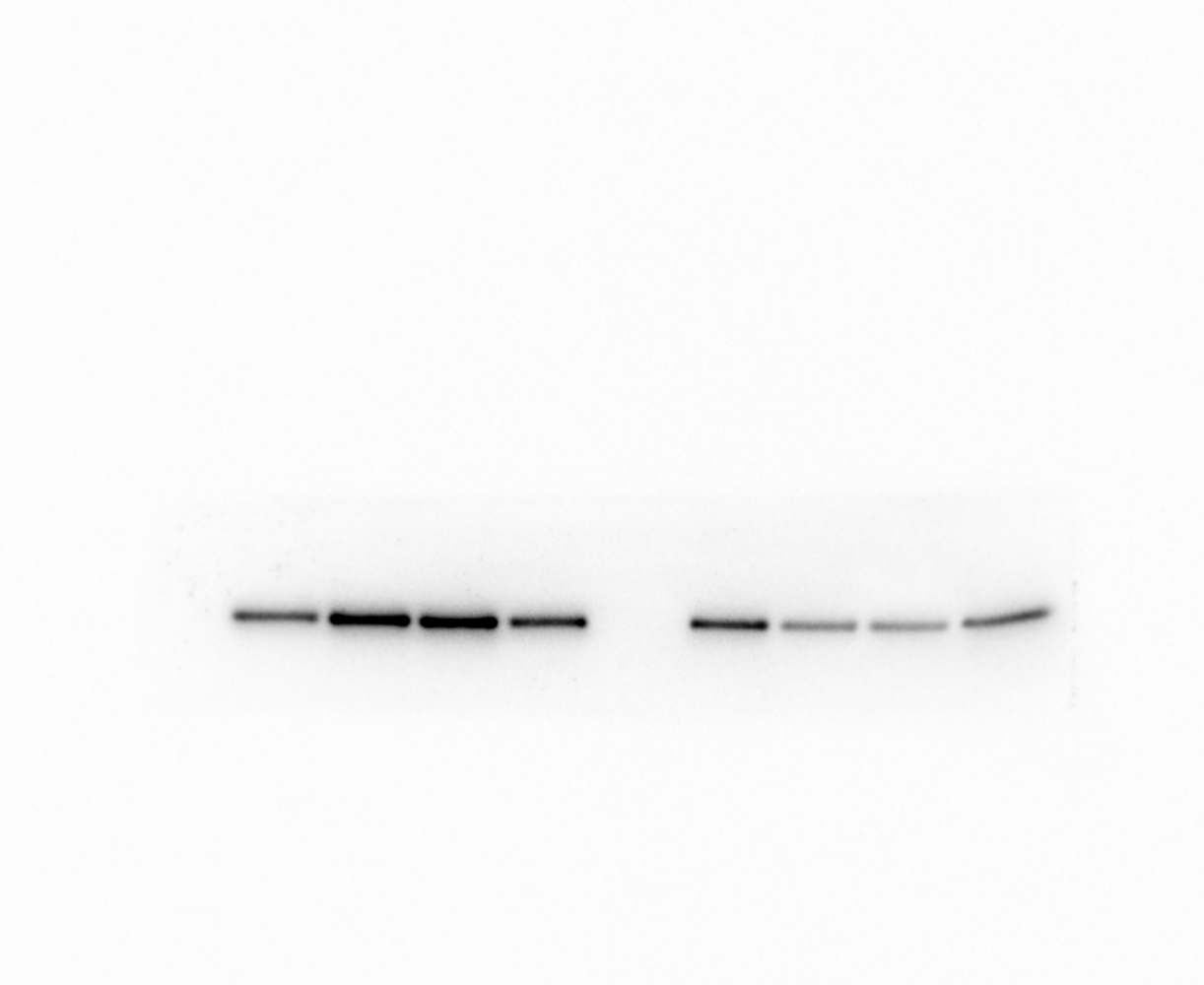


Fig.6F-PTEN


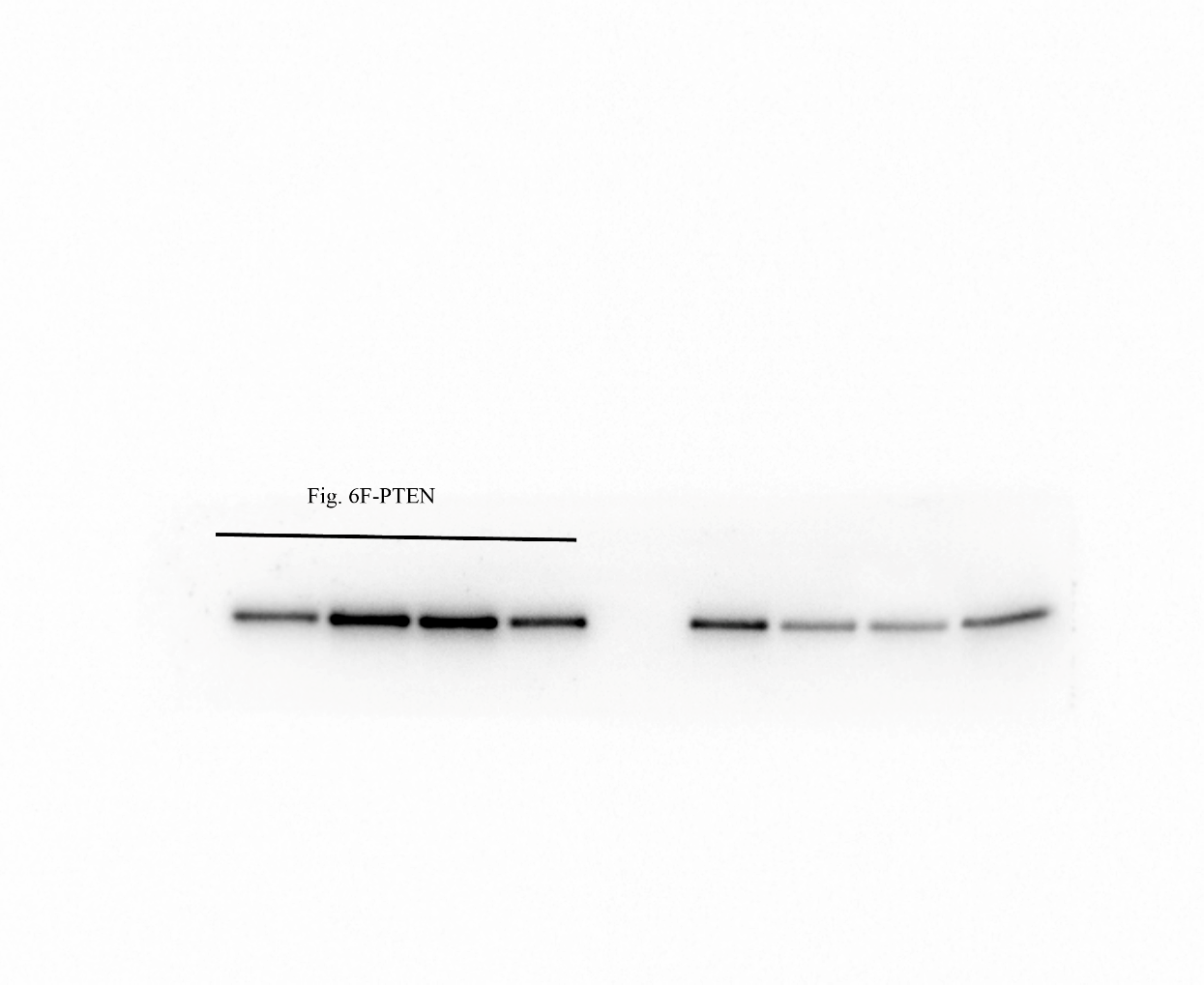


Fig.6F-PTEN-marked





Fig.7E-GAPDH





Fig.7E-PTEN
