# Supplementary material for: Exosomal circRNA BTG2 derived from RBP-J overexpressed-macrophages inhibits glioma progression via miR-25-3p/PTEN
Source: Cell Death Dis. 2022 May 28;13(5):506. doi: 10.1038/s41419-022-04908-4 (PMC9148311; doi:10.1038/s41419-022-04908-4)
Supplement: Supplementary file 2 — Supplemental material [file 41419_2022_4908_MOESM2_ESM.docx]

**Fig. S1**


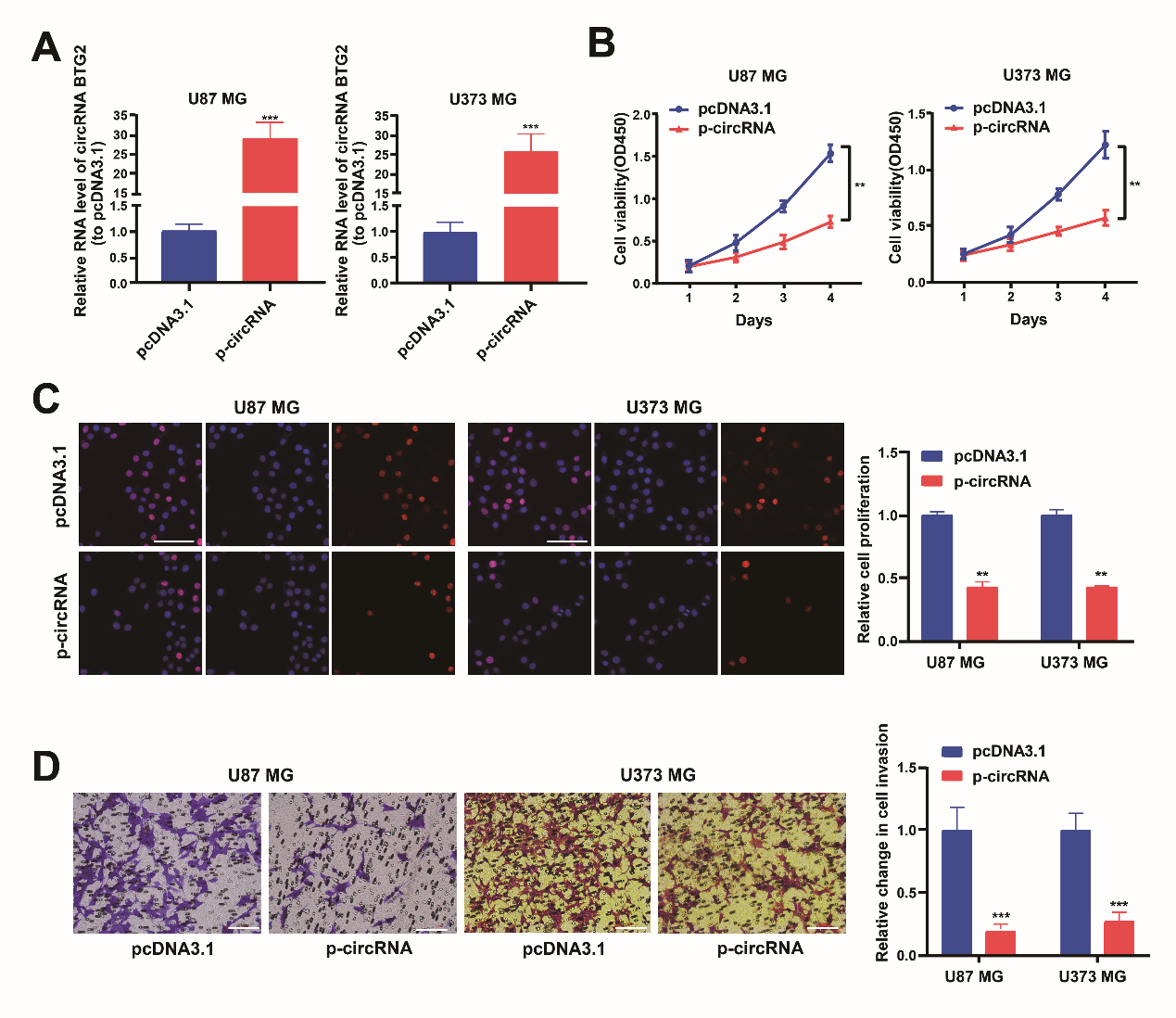


**Fig. S1. circRNA *BTG2* inhibits proliferation and invasion of glioma cells.** (A) U87 MG and U373 MG cells were transfected with circRNA *BTG2* overexpression plasmids or controls. (B, C) Cell proliferation in glioma cell lines U87 MG and U373 MG was assessed by CCK-8 assay (B) or EdU assay (C). (D) Cell invasion of glioma cell lines U87 MG and U373 MG was assessed by Transwell assay (bar=100μm). All experiments were performed three times. **P<0.01 and ***P<0.001 for statistical differences.

**Fig. S2**

**
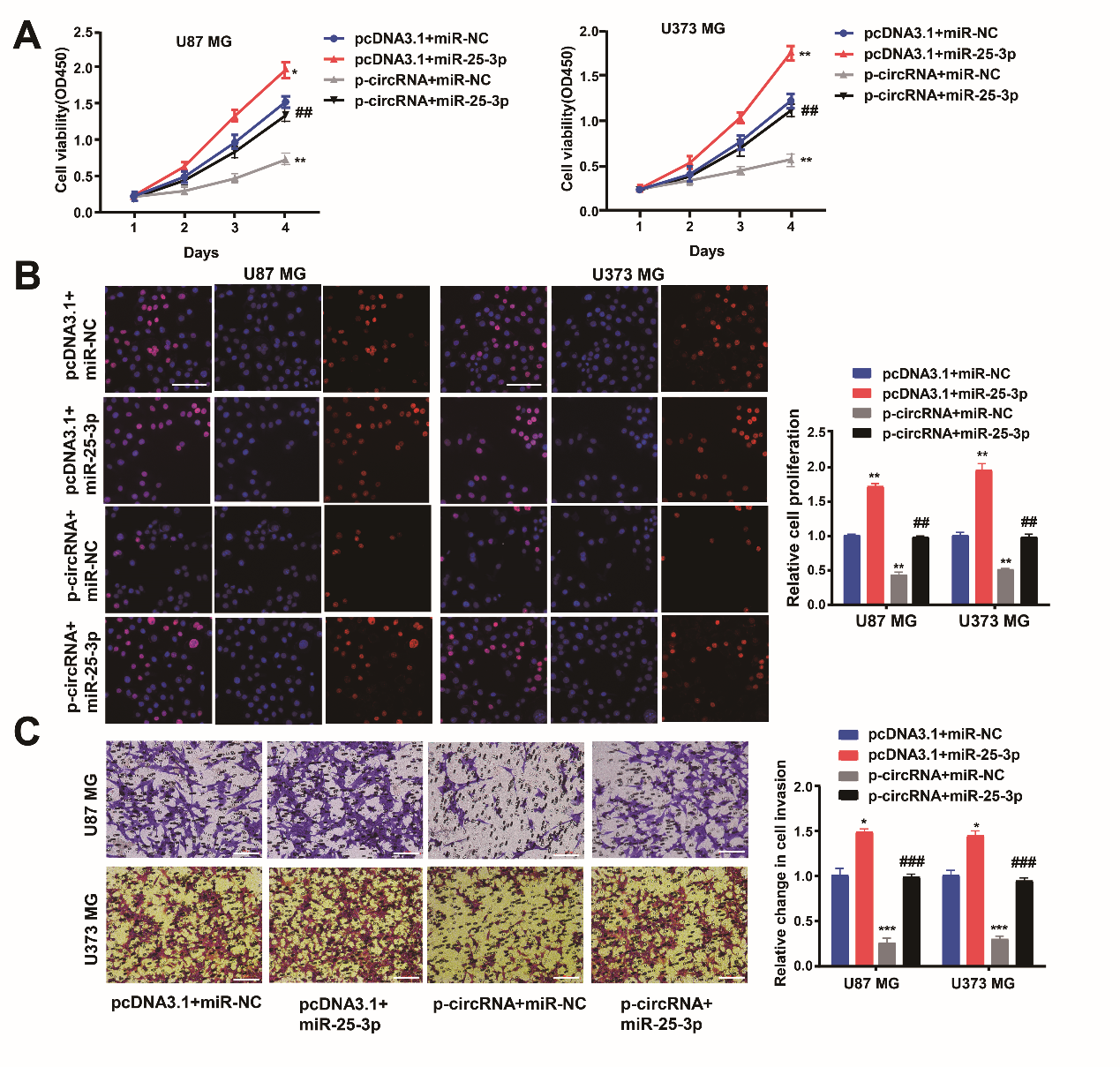
**

**Fig. S2. circRNA *BTG2* inhibits proliferation and invasion of glioma cells by sponging miR-25-3p.** (A, B) Cell proliferation in glioma cell lines U87 MG and U373 MG was assessed using CCK-8 assay (A) and EdU assay (B). (C) Cell invasion of glioma cell lines U87 MG and U373 MG was assessed by Transwell invasion assay (bar=100μm). All experiments were performed three times. *P<0.05, **, ##P<0.01 and ***, ###P<0.001 as indicated. * vs. pcDNA3.1+miR-25-3p NC, # vs. p-circRNA *BTG2*+miR-25-3p NC.
